# Polypeptide Nanogels With Different Functional Cores Promote Chemotherapy of Lung Carcinoma

**DOI:** 10.3389/fphar.2019.00037

**Published:** 2019-02-04

**Authors:** Kai Niu, Nan Li, Yunming Yao, Chunjie Guo, Yuanyuan Ge, Jianmeng Wang

**Affiliations:** ^1^Department of Otorhinolaryngology Head and Neck Surgery, The First Hospital of Jilin University, Changchun, China; ^2^Department of Neonatology, The First Hospital of Jilin University, Changchun, China; ^3^Department of Abdominal Ultrasound, The First Hospital of Jilin University, Changchun, China; ^4^Department of Radiology, The First Hospital of Jilin University, Changchun, China; ^5^Department of Geriatrics, The First Hospital of Jilin University, Changchun, China

**Keywords:** nanogel, reduction-responsive, controlled drug delivery, lung carcinoma, antitumor

## Abstract

Two kinds of tumor microenvironment-responsive polypeptide nanogels were developed for intracellular delivery of cytotoxics to enhance the antitumor efficacies and reduce the side effects in the chemotherapy of lung carcinoma. The sizes of both doxorubicin (DOX)-loaded nanogels methoxy poly(ethylene glycol)–poly(L-phenylalanine-*co*-L-cystine) [mPEG–P(LP-*co*-LC)] and methoxy poly(ethylene glycol)–poly(L-glutamic acid-*co*-L-cystine) [mPEG–P(LG-*co*-LC)] (NGP/DOX and NGG/DOX) were less than 100 nm, which was appropriate for the enhanced permeability and retention (EPR) effect. The bigger and smaller scale of nanoparticle could induce the elimination of reticuloendothelial system (RES) and decrease the *in vivo* circulating half-life, respectively. The loading nanogels were stable in the neutral environment while quickly degraded in the mimic intracellular microenvironment. Furthermore, the DOX-loaded reduction-responsive nanogels showed significantly higher tumor cell uptake than free DOX⋅HCl as time went on from 2 to 6 h. In addition, these DOX-loaded nanogels showed efficient antitumor effects *in vivo*, which was verified by the obviously increased necrosis areas in the tumor tissues. Furthermore, these DOX-loaded nanogels efficiently reduced the side effects of DOX. In conclusion, these reduction-responsive polypeptides based nanogels are suitable for the efficient therapy of lung carcinoma.

## Introduction

Lung carcinoma has been the most common malignancy in both men and women with high mortality (Kanodra et al., 2015; Kernstine, 2017). Chemotherapy, as a traditional treatment, is important to the treatment of lung cancer. However, the main therapeutic platforms available for chemotherapy drugs currently have the disadvantages of not being able to effectively aggregate in tumor cells and causing various systemic side effects. Due to these deficiencies, various nanomedicine delivery systems have been studied such as micelles (Feng et al., 2017; Shen et al., 2017; Xu et al., 2017; Kosakowska et al., 2018; Li et al., 2018b; Sun et al., 2018), nanogels (Ding et al., 2011; Guo et al., 2017, 2018; Jiang et al., 2018b; Li et al., 2018d; Zhang et al., 2018; Zhu et al., 2018), polymer–drug conjugates (Zhao et al., 2017; Cong et al., 2018; Li et al., 2018a; Yang et al., 2018), liposomes (Allen and Cullis, 2013; Zhao et al., 2013; Piffoux et al., 2018), and so forth (Ding et al., 2014; Tao et al., 2015, 2017; Chen et al., 2017a; Armstrong and Stevens, 2018; Ji et al., 2018; Jiang et al., 2018a; Li et al., 2018b,c; Xiao et al., 2018), which help to improve the drug accumulation in the tumor, to achieve the effective control of drug release in the tumor lesions, and to reduce the side effects (Yu et al., 2016; Zhang et al., 2017). Among them, nanogels have the strong core–shell structures by cross-linking, which enables them not only to exhibit high drug loading capacity, but also to prevent drug leakage (Ye et al., 2016; Chen et al., 2017b). In addition, the suitable sizes of the nanogels made them can efficiently accumulate at the tumor lesions by the enhanced permeability and retention (EPR) effect (Acharya and Sahoo, 2011). More importantly, additional functionality is given to crosslinkers to achieve “switch on/off” release of drugs from nanogels in tumor cells (Mura et al., 2013). In addition, the stability of nanogels is one of the major obstacles, which handers the *in vivo* application of them. Fortunately, the chemistry crosslinking has been widely used to enhance the stability and functionality of nanogels, which is more stable than non-covalent interactions (Ryu et al., 2010; Jiang et al., 2018a). Furthermore, cross-linking nanoparticles with different functional chemical crosslinkers could controllably release the laden drugs according to the redox, low pH, and high enzyme level of tumor microenvironments (Zha et al., 2011).

Due to the different metabolic pathways of tumor cells, the microenvironments of tumor tissues show hypoxia, low sugar, and low pH (Parks et al., 2013). It is worth noting that malignant cells show the reductive intracellular microenvironment (Ge and Liu, 2013). Therefore, the reduction-sensitive polymeric nanoparticles have attracted more and more attention in the realm of smart antitumor drug delivery (Cheng et al., 2013; Phillips and Gibson, 2014).

**Scheme 1:**
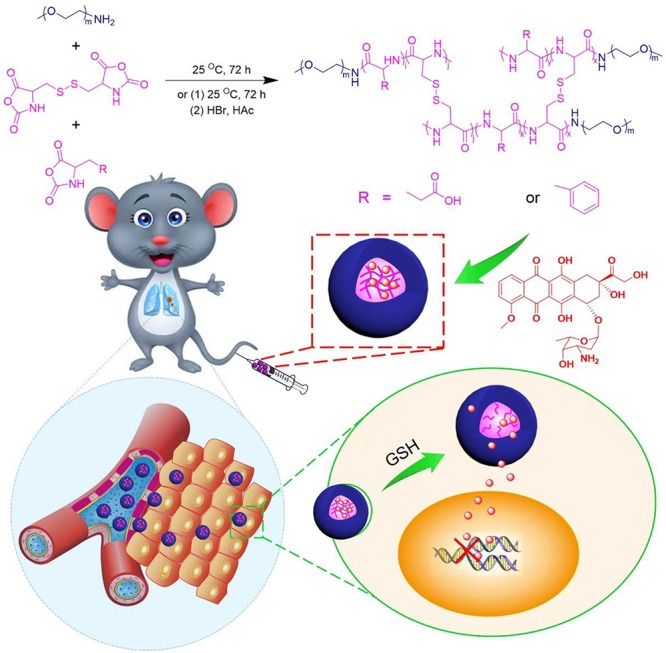
Synthetic pathway for mPEG–P(LP-*co*-LC) and mPEG–P(LG-*co*-LC) nanogel, illustrations of DOX encapsulation by nanogel, and its circulation, intratumoral accumulation, endocytosis, and targeting intracellular DOX release after intravenous injection.

Herein, we reported the drug delivery potential of reduction-sensitive polypeptide nanogels formulations, which could suppress lung carcinoma cell proliferation at low dose and reduce unwanted adverse effects. The reduction-responsive methoxy poly(ethylene glycol)–poly(L-phenylalanine-*co*-L-cystine) (mPEG–P(LP-*co*-LC)) and methoxy poly(ethylene glycol)–poly(L-glutamic acid-*co*-L-cystine) mPEG–P(LG-*co*-LC) nanogels were prepared to selectively deliver chemotherapy agents (Scheme 1). Typically, nanogels loaded with doxorubicin (DOX) were used as models for clinical antitumor drug. Results showed that both DOX-loaded nanogels exhibited satisfactory antitumor activity and higher safety than free DOX⋅HCl. These DOX-loaded nanogels are able to serve as satisfactory nanoplatforms for the therapy of lung carcinoma.

## Materials and Methods

Methoxy (ethylene glycol) (mPEG) was purchased from Aladdin Industrial Co., Ltd. (Shanghai, China) and mPEG-NH_2_ was synthesized by trimethylamine modified. L-Glutamic acid (LC), L-phenylalanine (LP), and L-cystine (LC) were obtained from GL Biochem, Ltd. (Shanghai, China). LG NCA, LP NCA, and LC NCA were prepared as reported in previous work (Huang et al., 2015; Shi et al., 2017). Doxorubicin hydrochloride (DOX⋅HCl) was bought from Beijing Huafeng United Technology Co., Ltd. (Beijing, China). Glutathione (GSH) (used for cell culture) was bought from Aladdin Reagent Co., Ltd. (Shanghai, China). Both 4,6-Diamidino-2-phenylindole (DAPI) and methyl thiazolyl tetrazolium (MTT) were obtained from Sigma–Aldrich (Shanghai, China). Hematoxylin and eosin were purchased from Merck Company (Darmstadt, Germany).

### Characterizations

Proton Nuclear Magnetic Resonance (^1^H NMR) spectra were detected on a Bruker AV 600 NMR spectrometer (Billerica, MA, United States) using deuterated dimethyl sulfoxide (DMSO-*d*_6_) as the solvent. Fourier-transform infrared spectroscopy (FT-IR) was performed on a Bio-Rad Win-IR instrument (Bio-Rad Laboratories Inc., Cambridge, MA, United States) by using potassium bromide method. The morphology of NGP/DOX and NGG/DOX nanogels were visualized on JEM-1011 transmission electron microscope (TEM; JEOL, Tokyo, Japan). Sizes of NGP/DOX and NGG/DOX were determined by dynamic laser scattering (DLS) measurement on a WyattQELS instrument (DAWN EOS, Wyatt Technology Corporation, Santa Barbara, CA, United States), and the scattering angle was set at 90°.

### Syntheses and Characterizations of mPEG–P(LP-*co*-LC) and mPEG–P(LG-*co*-LC)

The reduction-responsive mPEG–P(LP-*co*-LC) nanogel was synthesized through the one-step ROP of LP NCA and LC NCA with amino-terminated mPEG (mPEG–NH_2_) as a macroinitiator according to the reports in our previous works. Firstly, 2 g of mPEG-NH_2_, 2.3 g of NCA LP NCA and 0.9 g of LC NCA were dissolved in 100 mL of DMF and stirred for three days. The obtained solution was poured into 700 mL of the ethyl ether twice, and the white precipitate was collected. After vacuum drying, mPEG–P(LP-*co*-LC) was obtained with the yield of 75.2%. mPEG–P(BLG-*co*-LC) was prepared in a similar route. mPEG—P(LG-*co*-LC) was generated by removing the benzyl group from mPEG–P(BLG-*co*-LC). mPEG–P(BLG-*co*-LC) was dissolved in dichloroacetic acid (100 mg mL^-1^) and a 33 wt. % solution of HBr in acetic acid was added subsequently (20 mL for 1 g copolymer). After stirring for 1 h at 30°C, the mixture was precipitated into diethyl ether (10 times volume of the reaction solution). The obtained product was further dried under vacuum at room temperature for 24 h after washing twice with diethyl ether (Yield: 80.5%). The products were characterized by proton nuclear magnetic resonance (^1^H NMR), Fourier-transform infrared (FT-IR), inductively coupled plasma optical emission spectroscopy (ICP-OES), and elemental analysis.

### DOX Encapsulation

Nanogel was loaded with DOX by nanoprecipitation method. Firstly, 50 mg of nanogel was dispersed in 20 mL of N,N-dimethylformamide (DMF). After adding 10 mg of DOX⋅HCl, the solution was further stirred for 12 h at room temperature. Then, 2 mL of phosphate buffered saline (PBS; 0.01 M) was slowly mixed into the above solution, along with 18 mL of MilliQ water. The final mixture was dialyzed against MilliQ water for 24 h (molecular weight cut-off (MWCO) = 3500 Da) after stirring for 12 h at room temperature. The MilliQ water was changed every 2 h. Finally, NGP/DOX and NGG/DOX nanogels were obtained by filtration and lyophilization.

The drug loading content (DLC) and drug loading efficiency (DLE) were detected by standard curve method, using fluorescence spectroscopy on a Photon Technology International (PTI) Fluorescence Master System with Felix 4.1.0 software (PTI, Lawrenceville, NJ, United States; kex = 480 nm). The DLC and DLE of NGP/DOX and NG/DOX were calculated according to Eqs. (1) and (2), respectively.

$$\mathrm{DLC}(\%)\;=\;\frac{\mathrm{Weight}\;\mathrm{of}\;\mathrm{Drug}\;\mathrm{in}\;\mathrm{Nanogel}}{\mathrm{Weight}\;\mathrm{of}\;\mathrm{Drug}\;-\;\mathrm{Loaded}\;\mathrm{Nanogel}}\;\times \;100\%\;\;\;\;\;\;\;\;\;\;\;\;\;\;\;\;\;\;(1)$$

$$\mathrm{DLE}(\%)\;=\;\frac{\mathrm{Weight}\;\mathrm{of}\;\mathrm{Drug}\;\mathrm{in}\;\mathrm{Nanogel}}{\mathrm{Weight}\;\mathrm{of}\;\mathrm{Feeding}\;\mathrm{Drug}}\;\times \;100\%\;\;\;\;\;\;\;\;\;\;\;\;\;\;\;\;\;\;\;\;\;\;\;\;(2)$$

### *In vitro* DOX Release

*In vitro* drug release profiles of DOX from NGP/DOX and NGG/DOX nanogels were performed in PBS (pH 7.4) with or without 10 nM GSH. 10 mL of nanogel (0.1 mg mL^-1^) aqueous solution was transferred into an end-sealed dialysis bag (MWCO = 3500 Da). The release experiment was carried out by putting the end-sealed dialysis bag into the corresponding release medium (100 mL) at 37°C with continuous shaking at 75 rpm in the dark. At fixed time intervals, 2 mL of release medium was removed and an equal volume of fresh medium was added. The amount of released DOX was measured by the fluorescence spectrophotometer (kex = 480 nm).

### Cell Culture

Under the conditions of 37°C and 5% (V/V) carbon dioxide (CO_2_), the human lung Lewis cells were cultured in RPMI-1640, which was supplemented with 10% (V/V) fetal bovine serum (FBS), penicillin (100 IU mL^-1^), and streptomycin (100 IU mL^-1^).

### Intracellular DOX Release

The intracellular DOX release from NGP/DOX and NGG/DOX were measured by confocal laser scanning microscopy (CLSM) toward Lewis cells. The cells (15,000 cells) were seeded in disks, incubated in 1 mL of RPMI-1640 medium containing 10% FBS for 24 h, and pretreated with 10 mM GSH or buthionine sulfoximine (BSO) for 2 h. After removing the medium and subsequently washing three times with PBS (pH 7.4) solution, 1 mL of NGP/DOX and NGG/DOX solution in RPMI-1640 was added, with a final DOX dose of 10 μg mL^-1^. The cells treated with equivalent free DOX without GSH pretreatment were used as control. After another 2 h of incubation, the cells were washed with PBS for five times, and fixed with 4% (W/V) PBS-buffered paraformaldehyde at room temperature for 30 min. The cellular nuclei were then stained at 37^°^C for 3 min using DAPI. A CLSM (Carl Zeiss, LSM 780, Jena, Germany) was used to view the intracellular localization of DOX.

### Cytotoxicity Assays

The cytotoxicities of NGP/DOX, NGG/DOX and free DOX were evaluate din Lewis cells at different conditions. The cells were planted in 96-well plates (7 × 10^3^ cells per well) in 200 μL of RPMI-1640 medium supplemented with 1X penicillin/streptomycin and 10% fetal bovine serum. After incubation for 24 h at 37°C, the cells were pretreated with 10 mM GSH or BSO for 2 h. Subsequently, each culture medium was replaced by 180 μL of RPMI-1640 containing NGP/DOX, NGG/DOX and free DOX at equivalent concentrations, respectively, with the DOX ranging from 0.3 to 18.4 μM. After a further 24, 48, and 72 h incubation, 20 μL MTT (5 mg mL^-1^) in PBS was added to each well, followed by another 4°h incubation at 37°C. Then the sediment was dissolved in 150 μL DMSO after the medium was removed. The absorbance at 490 nm of the above solution was determined on an ELx808 microplate reader (Bio-Tek Instruments, Inc., Winooski, VT, United States). The percentage of cell viability was determined by comparing the absorbance of the sample cells and the control cells [Eq. (3)].

$$\mathrm{Cell}(\%)\;=\;\frac{A_{\mathrm{Sample}}}{A_{\mathrm{Control}}}\;\times \;100\%\;\;\;\;\;\;\;\;\;\;\;\;\;\;\;\;\;\;\;\;\;\;\;\;\;(3)$$

### Animal Procedures

5-Week-old male BALB/c mice weighting 18 ± 0.2 g were supplied by the Jilin University Experiment Animal Center (Changchun, China). All animal experiments were performed according to the Guidelines for Animal Care and Use of Jilin University. The tumor grafted mouse model was established by subcutaneous injection of 100 μL of cell suspension containing 2 × 10^6^ Lewis cells in PBS into the armpit of right anterior forelimb.

### *In vivo* Antitumor Assessments

The tumor volumes and mice’s body weights were monitored every two days from the second day after the inoculation of Lewis cells (that was, Day 1). When tumor volume increased to about 100 mm^3^ after 8 days of inoculation, the nude mice were randomly divided into 7 groups (*n* = 10), that was, free DOX, NGP/DOX or NGG/DOX at a DOX dose of 3 or 6 mg (kg BW)^-1^ and normal saline (control group). The formulations of DOX were recorded as DOX/3, DOX/6, NGP/DOX/3, NGP/DOX/6, NGG/DOX/3 and NGG/DOX/6, respectively. At the same time, the treatments began with injecting100 μL of normal saline and various DOX preparations in normal saline into the tail vein of mice for four times every 5 days. The tumor sizes were measured every day, and the body weights were measured every subsequent day. Tumor volumes [Eq. (4)] and body weights were used to evaluate the antitumor efficacy and security *in vivo*.

$$V({\mathrm{mm}}^{3})\;=\;\frac{L\;\times \;S^{2}}{2}\;\;\;\;\;\;\;\;\;\;\;\;\;\;\;\;\;\;\;\;\;\;(4)$$

In Eq. (4), L (mm) was the largest diameter of tumor, and S (mm) was the smallest diameter.

The following formula was used to calculate the tumor inhibition ratio:

$$\mathrm{Tumor}\;\mathrm{inhibition}\;\mathrm{rate}\;\left(\%\right)\;=\;(V_{\mathrm{control}\;}\;-\;V_{\mathrm{sample}})/V_{\mathrm{control}}\;\;\;\;\;\;\;\;\;\;\;\;\;\;\;\;\;\;\;\;\;\;\;\;\;(5)$$

In Eq. (5), *V*_control_ and *V*_sample_ represented the tumor volumes of control groups and sample groups, respectively.

The weights of the major organs were recorded. The organ indices of all the organs of mice were calculated by [Eq. (6)].

$$\mathrm{Organ}(\%)\;=\;\frac{W_{\mathrm{Organ}}}{W_{\mathrm{Body}}}\;\times \;100\%\;\;\;\;\;\;\;\;\;\;\;\;\;\;\;\;\;\;\;\;\;(6)$$

### Immunohistochemical Analyses of Tumor Tissues

On day 27, the Lewis lung carcinoma-grafted BALB/c mice were killed by cervical dislocation 5 days after the last injections. The tumors and major organs (heart, liver, spleen, lung, kidney, thymus, and marrow) were isolated at first, and then fixed with 4% (W/V) paraformaldehyde overnight, followed by dehydration, clearing, wax infiltration, and embedding. The paraffin-embedded tumors and organ tissues were cut at a thickness of 5 μm for hematoxylin and eosin (H&E) staining. Paraffin sections with a thickness of 3 μm were used for immunohistochemical staining (including caspase-3, survivin, Bax, and Bcl-2) to assess the pathological and immunological characteristics of tumor tissues. The instruments used included Leica RM 2245 paraffin machine (Leica, Germany), Leica HI1210 fishing machine (Leica, Germany), Leica EG1150H embedding machine (Leica, Germany), Leica HI1220 booth machine (Leica, Germany), Olympus BX51 microscope (Olympus, Japan), and Motic image analysis system (Motic Industrial Group Co., Ltd., Xiamen, China).

### Histopathological and Biochemical Analyses of Organs

The major internal organs and tissues (heart, liver, spleen, lung, kidney, thymus, and marrow) were collected at the same time. The organs from healthy mice were used as controls. All the organs involved were divided into two parts as follows: (i) one part (excluding marrow) fixed with 4% (*W*/*V*) PBS-buffered paraformaldehyde was prepared for the histopathological analyses by H&E staining. (ii) The other part was used to detect the organ function-related biochemical indicators, including blood urea nitrogen (BUN), creatinine (Cr), alanine aminotransferase (ALT), aspartate aminotransferase (AST), creatine kinase (CK), creatine kinase-MB (CK-MB) and lactate dehydrogenase (LDH), by commercial enzyme-linked immunosorbent assay (ELISA) kits. The biochemical indicators in serum were also tested. Briefly, 300 μL of blood without anticoagulant was centrifuged at 3000 rpm for 10 min. The serum was then collected to detect the clinical biochemical parameters. The data from healthy nude mice were used as controls. The histopathological results were detected and analyzed by Olympus BX51 microscope and Motic image analysis system, respectively.

### Detections of White Blood Cell (WBC) Count and Bone Marrow Cell Micronucleus Rates (BMMRs)

On day 27, 20 μL of blood (anticoagulated through enucleation method) was taken from each nude mouse to count the WBCs. The sternums from BALB/c mice were decalcified and fixed for 10 days after being placed in 10% (V/V) formic acid-formalin solution. The data from normal nude mice were used as controls. Then, the tissues were dehydrated, cleared, wax infiltrated, and embedded. For each sternum, paraffin sections with a thickness of 5 μm were collected for H&E staining, with an interval of 50 μm. The H&E-stained section was used to evaluate the BMMR.

### Statistical Analyses

All tests were carried out at least three times, and the relevant data were expressed as mean ± standard deviation (SD). Statistical analysis was performed using SPSS 13.0 statistical software (SPSS Inc., Chicago, IL, United States), ^∗∗∗^*P* < 0.05 was considered statistically significant, and ^∗∗^*P* < 0.01 and ^∗^*P* < 0.001 were as considered significant differences.

## Results and Discussion

### Characterizations of NGP/DOX and NGG/DOX

**Figure 1:**
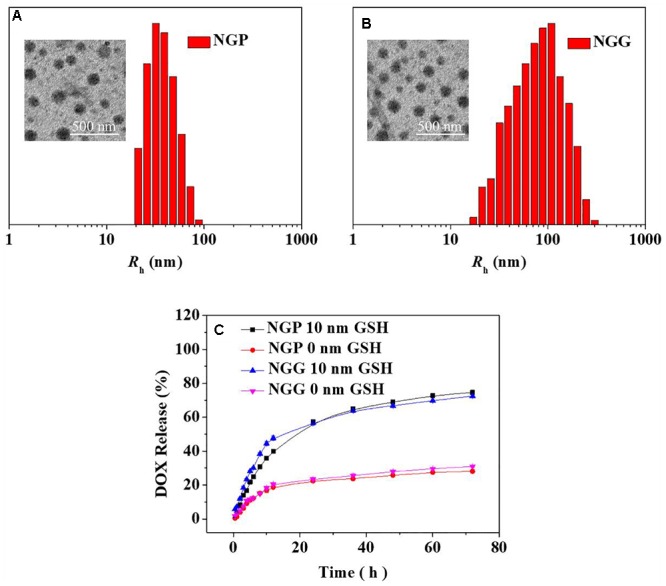
Chemo-Physical Characterizations. Typical TEM micrograph and *R*_h_ detected by DLS of NGP/DOX **(A)** and NGG/DOX **(B)**. *In vitro* DOX release from NGP/DOX and NGG/DOX **(C)** in PBS of pH 7.4 and 7.4 with 10 nm GSH at 37°C. Scale bar in B represents 500 nm. The statistical data are represented as mean ± standard deviation (*SD*; *n* = 3).

These reduction-responsive nanogels consisted of mPEG (hydrophilic shell) and disulfide-cross-linked P(LP-*co*-LC) or P(LG-*co*-LC) (hydrophobic core). The disulfide bond of the LC segment endowed the nanogels with reduction-responsiveness (Huang et al., 2015; Shi et al., 2017). These DOX-loaded nanogels were prepared through a modified nanoprecipitation method by adding DOX aqueous solution to dimethylformamide (DMF) solution containing NGP or NGG nanogel (Scheme 1) (Ding et al., 2013a; Huang et al., 2015; Liu et al., 2015; Shi et al., 2017). The drug loading content (DLCs) and drug loading efficiency (DLEs) of NGP/DOX and NGG/DOX were calculated to be 9.8, 54.7 and 15.2, 91.5 wt.%, respectively. As shown in the TEM images, NGP/DOX, and NGG/DOX showed a spherical morphology at a diameter of about 80 and 93 nm, respectively (Figure 1A,B). The *R*_h_ of NGP/DOX and NGG/DOX detected by dynamic laser scattering (DLS) were 78.1 ± 3.5 and 93.3 ± 3.5 nm, respectively (Figure 1A,B). The hydrodynamic size of NGP/DOX and NGG/DOX was slightly larger than the diameter detected by TEM due to the swelling of nanogels in the aqueous condition (Huang et al., 2015; Shi et al., 2017). Appropriate size facilitated the efficient accumulation of nanogels in the tumors (Ding et al., 2013b,c, 2015).

### *In vitro* Release Performance and Tumor Cell Inhibition

The DOX release performances of NGP/DOX and NGG/DOX were carried out in PBS with 0 or 10.0 mM glutathione (GSH). As shown in Figure 1C, during the 72 h test, only around 30% of DOX was released in the PBS with 0 mM GSH. In contrast, an obviously increased DOX release was observed in the PBS with 10.0 mM GSH. In detail, the proportions of cumulative released DOX from NGP/DOX and NGG/DOX in the initial 12 h were 47.8 and 39.8% in PBS with GSH (GSH+), respectively. After 72 h of incubation, the DOX released from NGP/DOX and NGG/DOX in the GSH+ medium (72.4 and 74.7%) were more than twice that in the GSH- medium (31.0 and 28.1%), respectively. The accelerated release of DOX should be due to GSH breaking the disulfide bond of nanogels (Huang et al., 2015). After 24 h, the drug release of nanogels was relatively decelerated and sustained. The release results indicated the NGP/DOX and NGG/DOX could efficiently release the DOX in tumor cells according to the different redox potential between intracellular and extracellular microenvironments, which might have more obvious antitumor activity.

**Figure 2:**
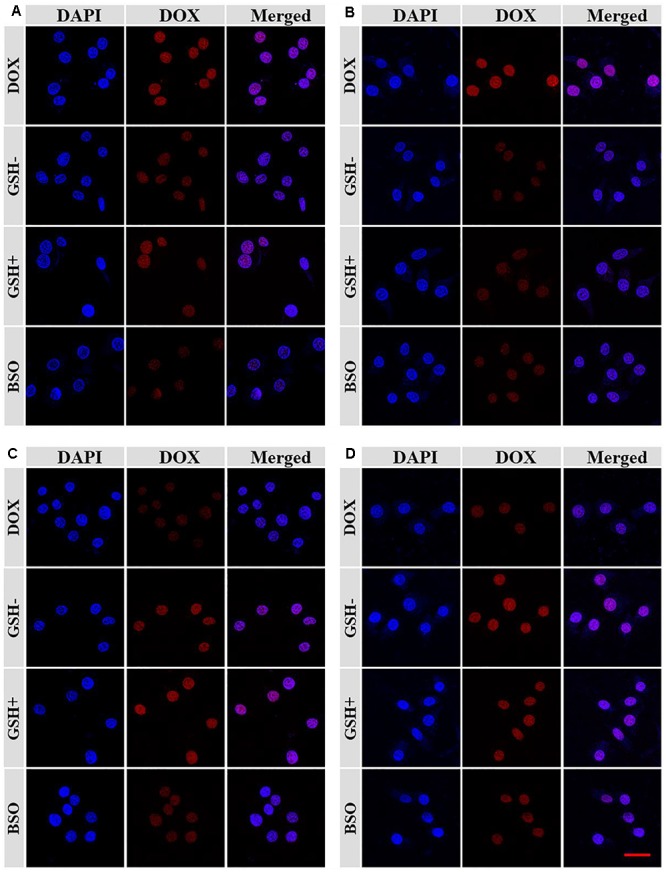
The CLSM microimages analyses for cell internalization of NGP **(A,C)** and NGG **(B,D)** after incubation with Lewis cells for 2 h **(A,B)** and 6 h **(C,D)**. Scale bar =50 μm.

Confocal laser scanning microscopy (CLSM) assays were performed toward Lewis lung carcinoma cells pretreated without (GSH-), with 10.0 mM GSH (GSH+) or buthionine sulfoximine (BSO) to verify the satisfactory intracellular DOX release of NGP/DOX and NGG/DOX. NG/DOX with a dosage of 10.0 μg mL^-1^ DOX⋅HCl was used to co-cultured with the GSH- or GSH+ cells for 2 and 6 h. The unpretreated cells co-cultured with equivalent free DOX⋅HCl were prepared as a control. As shown, the DOX fluorescence was shown in all the Lewis lung carcinoma cells treated with DOX formulations (Figure 2). As time went on, the DOX fluorescence in the nanogels with GSH pretreatment groups were higher than that of the free DOX⋅HCl and BSO pretreated groups. As far as we knew, only the DOX released from nanogels in the cells could be detected by CLSM (Ding et al., 2013c). These results verified the efficient endocytosis as well as the reduction-responsive intracellular DOX release of NGG/DOX and NGP/DOX. These results might be related to that with the pretreatment of GSH, the intracellular GSH content was increased, which facilitated the rapid release of DOX from nanogels. Moreover, the results were consistent with the *in vitro* DOX release kinetics in PBS (Figure 1C). It was interesting that the tumor cells incubated with free DOX⋅HCl exhibited the strongest DOX fluorescence at 2 h. This result was related to the way of free DOX⋅HCl entered cells was diffusion, which was faster than the endocytosis of nanogels (Ding et al., 2013b,c).

**Figure 3:**
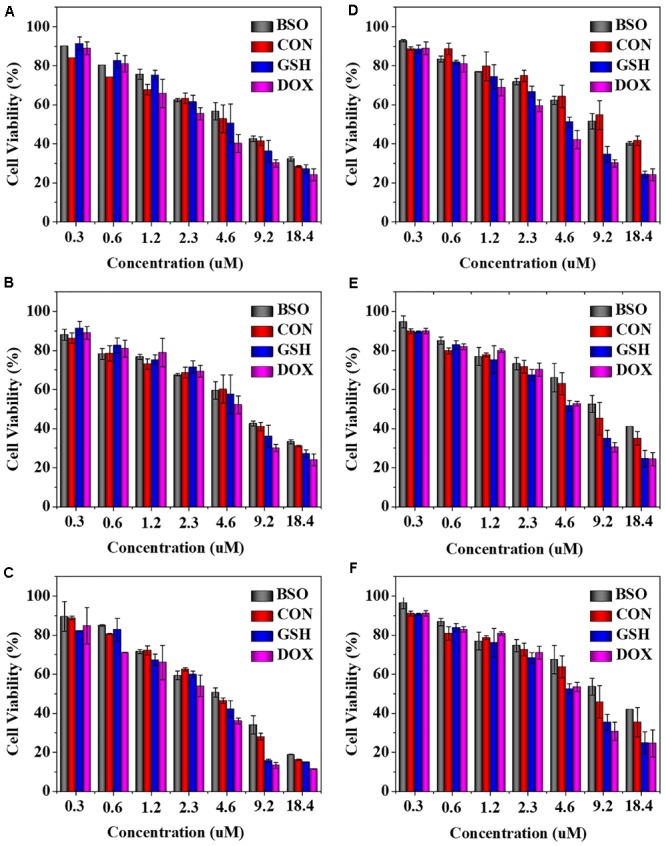
*In vitro* cytotoxicity of NGP **(A–C)** and NGG **(D–F)** after incubation with Lewis cells for 24, 48, and 72 h. The statistical data are presented as a mean ± *SD* (*n* = 6).

The *in vitro* antitumor activities of NGG/DOX and NGP/DOX were estimated by MTT assay at 24, 48, and 72 h. As shown in Figure 3, compared with the BSO pretreated and unpretreated groups, GSH pretreated nanogels showed obviously higher growth inhibition efficiency in Lewis cells during the test time. This more satisfactory inhibition of GSH pretreated nanogels might be related to the intracellular GSH content was increased after GSH pretreatment, which facilitated the rapid release of DOX from nanogels and showed stronger tumor killing effect. As time went on, the half maximal inhibitory concentrations (IC_50_s) of NGP/DOX decreased, but the order was constant: BSO < GSH- < GSH+. At 72 h, the IC_50_s of BSO, GSH-, and GSH+ pretreated groups were calculated to be 3.78, 3.30, and 2.61 μM mL^-1^, respectively. The NGG/DOX groups showed similar results, and the IC_50_s of BSO, GSH-, and GSH+ pretreated groups were calculated to be 12.12, 7.92, and 4.65 μM mL^-1^ at 72 h. The NGP/DOX and NGG/DOX groups pretreated by GSH had the lowest IC_50_, demonstrating their enhanced suppressor capability against the tumor cells and their advantage as potential antitumor drug formulations.

### *In vivo* Antitumor Efficacy

**Figure 4:**
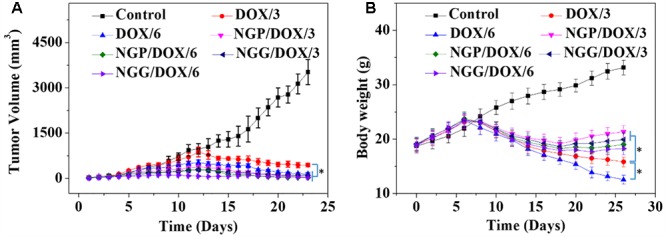
Tumor volume **(A)** and body weight **(B)** changes of Lewis lung carcinoma-bearing BALB/c mice in the course of treatment with control, or free DOX⋅HCl, or NGP/DOX, or NGG/DOX at a dosage of 3.0 or 6.0 mg DOX⋅HCl equivalent per kg body weight. Each set of data is represented as mean ±*SD* (*n* = 10; ^∗^*P* < 0.001).

**Figure 5:**
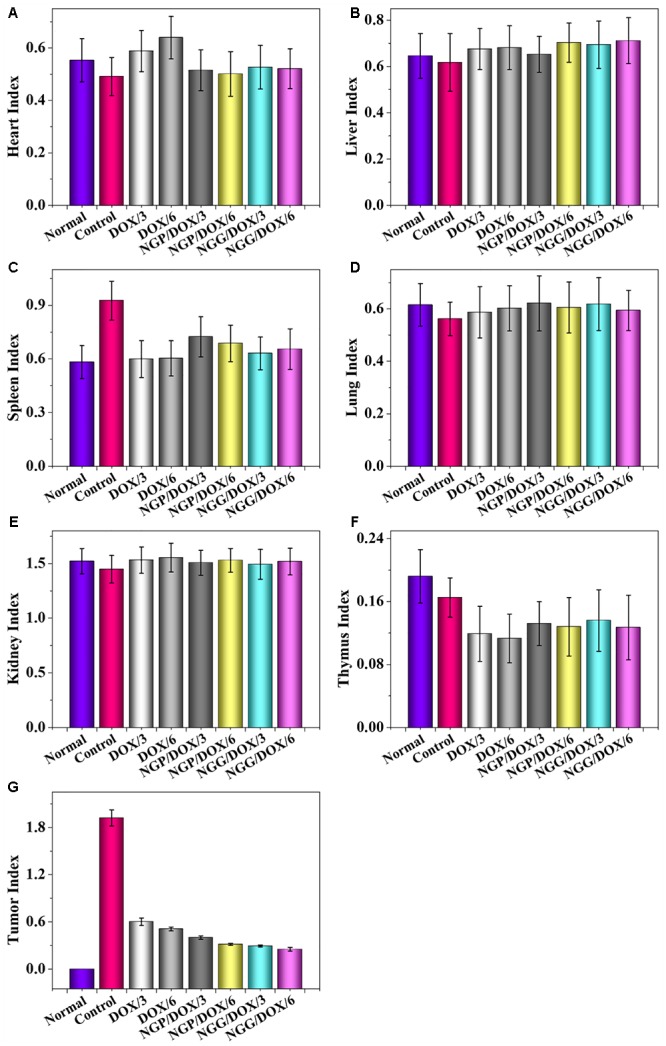
Evaluations of heart **(A)**, liver **(B)**, spleen **(C)**, lung **(D)**, kidney **(E)**, thymus **(F)**, and tumor **(G)** indexes of Lewis lung carcinoma-allografted mice after injected with NS, DOX/3, DOX/6, NGP/DOX/3, NGP/DOX/6, NGG/DOX/3, or NGG/DOX/6. Each set of data is represented as mean ±*SD* (*n* = 8).

The most important indicators of drug use *in vivo* were efficacy and safety, which must be rigorously evaluated before any newly formulations could be used clinically. Tumor-grafted animal models are the main method to evaluate the antitumor activities of drug delivery systems. As shown, compared with free DOX⋅HCl, DOX-loaded nanogels exhibited stronger antitumor effect (Figure 4A). This result was related to two factors: one was related to the EPR effect (Huang et al., 2015; Shi et al., 2017), which resulted more DOX-loaded nanogels accumulated in tumor sites, the other was due to the more controlled release of DOX from nanogels, which resulted less DOX was released in circulation and more DOX accumulated in tumor sites. The tumor inhibition rates of NGP/DOX/3, NGP/DOX/6, NGG/DOX/3, and NGG/DOX/6 groups were 98.9 ± 0.2%, 99.4 ± 0.03%, 97.15 ± 0.4%, and 99.1 ± 0.1%, which were much stronger than that of DOX/3 and DOX/6 groups (i.e., 87.4 ± 2.1% and 95.8 ± 3.0%; *P <* 0.001). Despite DOX also showed anti-tumor effect, the weight loss of mice was very obvious during the therapy (Figure 4B), especially in the DOX/6 group, indicating the severe systemic toxicity of DOX. In contrast, the groups treated with DOX-loaded nanogels showed tiny body weight loss, indicating the effective attenuation effect DOX-loaded nanogels. Furthermore, organ indices were provided to offer a general impression of the system toxicity of DOX. The tumor index was also calculated. As shown, the organ indices exhibited no obvious difference except the tumor indices (Figure 5), indicating these nanogels would efficiently suppress the tumor growth while not cause systemic toxicities *in vivo*.

**Figure 6:**
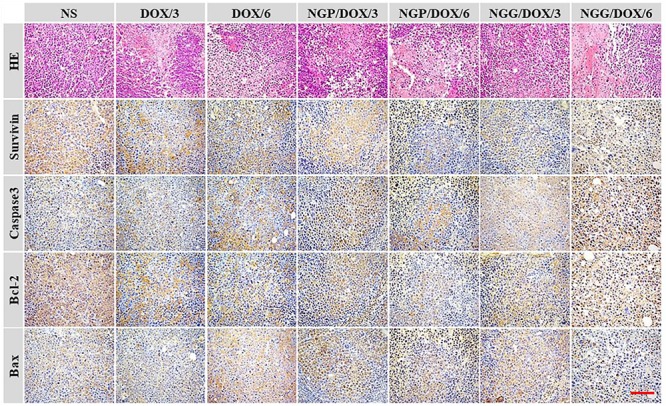
Immunohistochemical (i.e., caspase-3, survivin, Bax, and Bcl-2) analyses of tumor tissue sections after treatments with NS, free DOX⋅HCl at a dose of 3 and 6 mg (DOX/3, DOX/6), NGP/DOX, and NGG/DOX at a dose of 3.0 or 6.0 mg DOX⋅HCl equivalent per kg body weight. Scale bar = 50 μm, magnification: 200×.

In addition, the histopathological and immunohistochemical tests were performed to further confirm the antitumor efficacy of DOX-loaded nanogels (Figure 6). In this study, four immunohistochemical stainings caspase-3, survivin, Bax, and Bcl-2 were performed (Figure 6). It is well known that the process of cell apoptosis is regulated and controlled by various apoptotic genes. Notably, the caspase family plays a crucial role in the process of apoptosis (Huang et al., 2015; Shi et al., 2017). The activation of apoptosis-inducing factor caspase-3 is the key pathway for a variety of stimuli-induced apoptosis (Huang et al., 2015; Shi et al., 2017). As shown, the signals of pro-apoptotic protein Bax (brown) and caspase-3 (brown) of the nanogels treatment groups were much stronger than those of the free DOX⋅HCl treatment groups. In contrast, the anti-apoptotic protein Bcl-2 (brown) showed an obvious decline in the nanogels treatment groups. Furthermore, survivin was also tested to evaluate the cell survival (Huang et al., 2015; Shi et al., 2017). As shown, the signals of survivin (brown) were obviously reduced in the DOX-loaded nanogels treatment groups. These results fully verified that our DOX-loaded nanogels, especially NGP/DOX/6 and NGG/DOX/6, could be served as efficient nano-therapeutic agents.

As shown in Figure 6, H&E staining showed universal mitosis and mild hemorrhagic necrosis in the control group, indicating the rapid cell growth. In contrast, all the DOX formulations treatment groups showed varying degrees of tumor growth suppression. Specifically, DOX formulations caused a reduction in mitosis and extensive hemorrhage and necrosis. The treatment groups were ranked as follows according to the relative amount of necrotic tissues: NGP/DOX/6.0 > NGG/DOX/6.0 > DOX/6.0 > NGP/DOX/3.0 > NGG/DOX/3.0 > DOX/3.0. Furthermore, the results of semi-quantitative in Figure 10C,F showed the necrotic areas of NGP/DOX/6 and NGP/DOX/6 treatment groups were 1.4 and 1.2 times larger than those of the free DOX⋅HCl/6 treatment group, respectively.

### *In vivo* Security Evaluation

In this study, systematic safety was evaluated by monitoring the physical conditions and body weights changes, by analyzing the pathological morphology of various organs, by detecting the biological parameters from organs and serum, and by examining the BMMR and WBC levels after therapeutics. The *in vivo* systematic toxicity of DOX was reflected by body weight and histopathology of organs. Similar body weight gain trends were observed in each group of nude mice within the initial 1–10 days (Figure 4B). After that, the body weights of the nanogels treatment groups still showed similar growth trends on day 11–26. This result might be related to that controlled release of nanogels, which resulted less DOX release in the circulation. On the contrary, the body weights of free DOX⋅HCl treated groups showed significant downward trends within the same time interval, and larger doses cause more weight loss (*P* < 0.001). Especially the dose of 6.0 mg (kg BW)^-1^ DOX treatment group caused severe weight loss, indicating the toxicity of DOX were dose-dependent.

**Figure 7:**
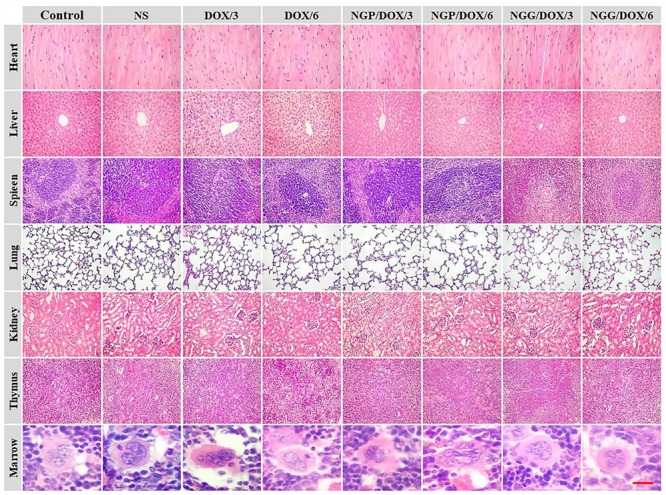
*Ex vivo* histopathological analyses (i.e., H&E) of lung tumor, visceral organ, thymus, and marrow sections after all treatments with control, NS, free DOX 3 and 6 mg (DOX/3, DOX/6), NGP/DOX, and NGG/DOX at a dose of 3.0 or 6.0 mg DOX⋅HCl equivalent per kg body weight (NG/DOX/3, NG/DOX/6). Magnification: 200×.

The histopathological analyses of major organs were shown in Figure 7. Significant neutrophil accumulation and myocardial fiber breakage were observed in the showed in the free DOX⋅HCl treatment groups. In contrast, the neutrophil accumulation did not occur in the nanogels treatment groups, the myocardial cells were arranged orderly, and the sarcolemma-maintained integrity, probably relate to the reduced accumulation of free DOX⋅HCl in heart. Moreover, the microregional necrosis of hepatocytes in the free DOX⋅HCl treatment group indicated that free DOX⋅HCl had significant hepatotoxicity. On the contrary, less structural interferences were observed in the nanogels treatment groups. Moreover, the nephrotoxicity of free DOX⋅HCl was also reduced by the nanogels, which was verified by the intact structure of the kidneys in the nanogels treatment groups. These results indicated that the DOX-loaded nanogels could effectively reduce systematic toxicity, probably due to the satisfactory stability of DOX-loaded nanogels. All the results confirmed that the DOX-loaded nanogels had good biocompatibility.

**Figure 8:**
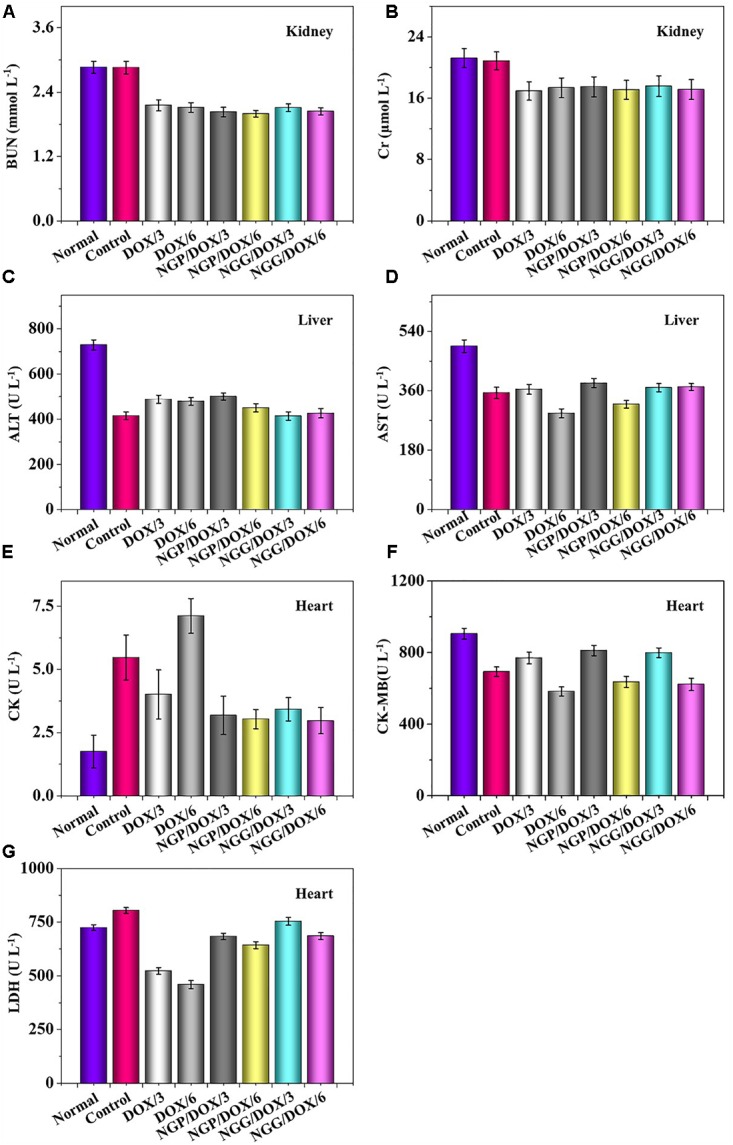
Biochemical parameter assays for safety evaluation. Evaluations of BUN **(A)**, Cr **(B)**, ALT **(C)**, AST **(D)**, CK **(E)**, CK-MB **(F)**, and LDH **(G)** in corresponding internal organs of normal mice or Lewis lung carcinoma-allografted BALB/c mice after treatment with NS, free DOX⋅HCl at a dose of 3 and 6 mg (DOX/3, DOX/6), NGP/DOX, and NGG/DOX at a dose of 3.0 or 6.0 mg DOX⋅HCl equivalent per kg body weight. Each set of data is represented as mean ±*SD* (*n* = 3).

**Figure 9:**
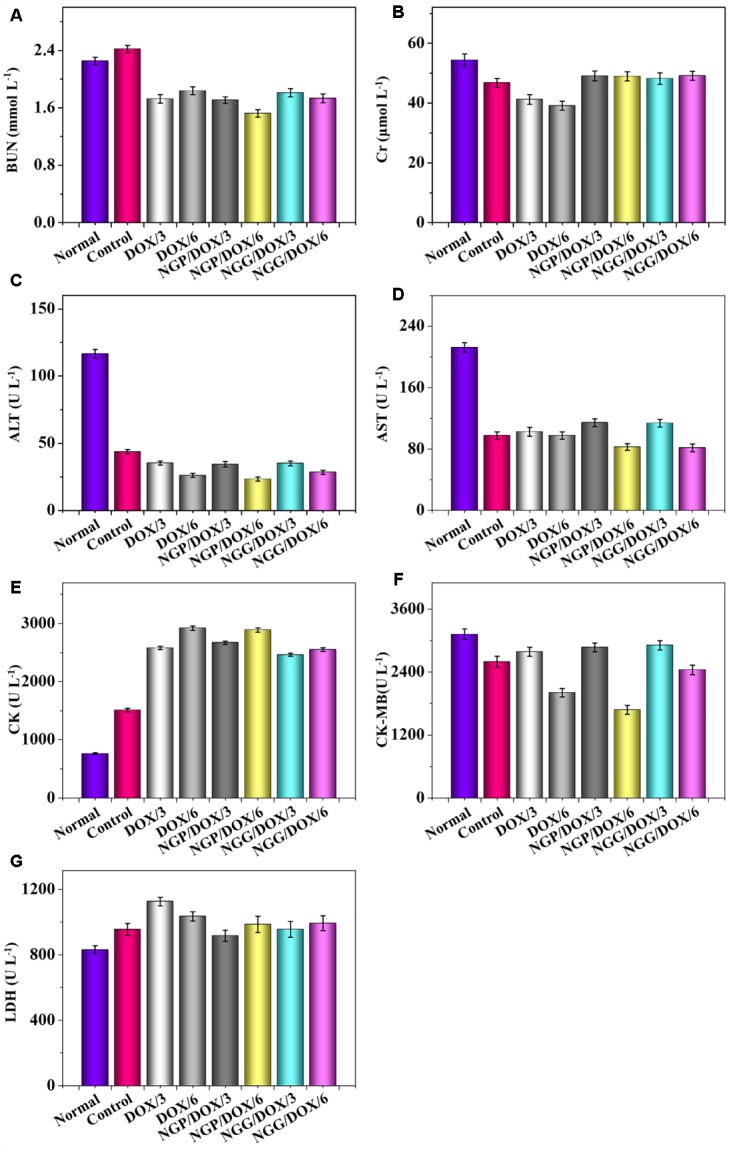
Biochemical parameter assays for safety evaluation. Evaluations of BUN **(A)**, Cr **(B)**, ALT **(C)**, AST **(D)**, CK **(E)**, CK-MB **(F)**, and LDH **(G)** in serum of normal mice or Lewis lung carcinoma-allografted BALB/c mice after treatment with NS, free DOX⋅HCl at a dose of 3 and 6 mg (DOX/3, DOX/6), NGP/DOX, and NGG/DOX at a dose of 3.0 or 6.0 mg DOX⋅HCl equivalent per kg body weight. Each set of data is represented as mean ±*SD* (*n* = 3).

The relevant clinical parameters of heart (CK, CK-MB, and LDH), liver (ALT and AST), and kidney (BUN and Cr) can reflect the function of corresponding organs. In this work, these clinical parameters were detected to verify the safety of DOX-loaded nanogels *in vivo*. As shown, the parameters of all organs except heart in each treated group were within the normal range (Figure 8). Free DOX⋅HCl/6 could cause serious damage to the heart (Figure 8E). The relevant parameters in serum were also tested by the commercial ELISA kits. All the clinical parameters of mice treated with DOX-loaded nanogels were equal or lower than normal nude mice treated with NS except CK (Figure 9). These results indicated that DOX-loaded nanogels did not cause serious organ dysfunction.

**Figure 10:**
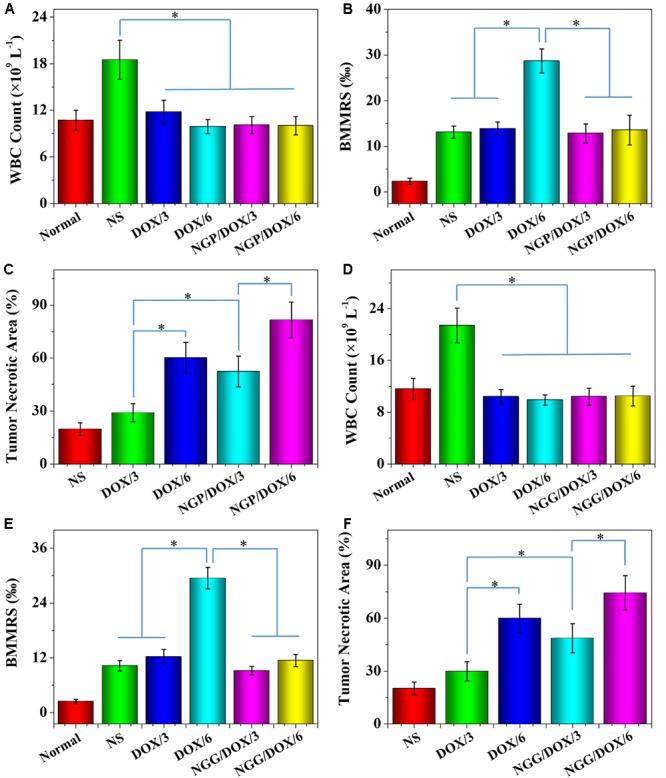
WBC counts **(A,D)**, BMMRs **(B,E)** and tumor necrotic area from H&E **(C,F)**, normal mice (as control), or Lewis lung carcinoma-allografted BALB/c nude mice after treatment with NS, or free DOX⋅HCl or NGP/DOX **(A–C)** or NGG/DOX **(D–F)** at a dose of 3.0 or 6.0 mg DOX⋅HCl equivalent per kg body weight. Normal BALB/c nude mice serve as controls (Normal). Each set of data is represented as mean ± SD (*n* = 10; ^∗^*P* < 0.001).

The number of WBC was tested to reflect the influence of chemotherapy drugs on the immune system. As shown in Figure 10A,D, the WBC counts of the NS treatment group was increased obviously. On the contrary, the DOX formulation treatment groups did not show increased WBC counts. The results indicated that the treatments with DOX formulations could effectively reduce the inflammation induced by tumor (*P* < 0.01).

The genotoxicity caused by chemotherapy drugs can be quantified by BMMR (Wang et al., 2018). As shown in Figure 10B,E, H&E-stained marrow sections were used to observe bone marrow mononuclear cells. These histopathological sections were also used to calculate BMMRs. Compared with normal mice, the BMMRs increased obviously in Lewis lung-grafted mice. For the mice treated with DOX formulations, a dose-related increase in BMMR was observed. Moreover, the BMMRs of the groups treated with free DOX⋅HCl were significantly greater than those of groups treated with DOX-loaded nanogels (*P* < 0.001). These results demonstrated that the physiological damage caused by free DOX was dose-dependent. Fortunately, DOX-loaded nanogels could effectively mitigate this injury.

## Conclusion

In this work, reduction-responsive DOX-loaded nanogels (NGP/DOX and NGG/DOX) were prepared by classical nanoprecipitation method. *In vitro* studies showed that both NGP/DOX and NGG/DOX groups exhibited stronger cellular uptake of Lewis cells compared with free DOX treatment groups. Furthermore, all the NGP/DOX and NGG/DOX groups exhibited more efficient antitumor efficacy than the free DOX⋅HCl treatment groups in the Lewis lung carcinoma grafted nude mouse model. Importantly, all the DOX-loaded nanogels could significantly reduce the systemic toxicity of DOX. Therefore, these polypeptides nanogels with high systemic safety could serve as promising nanodrug delivery platforms for the future lung carcinoma chemotherapy.

## Author Contributions

All authors conceived and designed the study. KN, NL, and YY performed the experiments. KN, NL, YY, CG, YG, and JW analyzed and interpreted the data, and prepared the manuscript.

## Conflict of Interest Statement

The authors declare that the research was conducted in the absence of any commercial or financial relationships that could be construed as a potential conflict of interest.
